# Identification of SARS-CoV-2 on the ocular surface in a cohort of COVID-19 patients from Brazil

**DOI:** 10.1177/15353702211024651

**Published:** 2021-07-19

**Authors:** Matheus S Gasparini, Leandro M dos Santos, Ahmad MA Hamade, Luísa G Gross, Arthur P Favarato, José PC de Vasconcellos, Mônica B de Melo, Pierina L Parise, Camila L Simeoni, Natália B Silva, Marcelo A da Silva Mori, André S Vieira, Alessandro dos Santos Farias, Fabiana Granja, Angelica Z Schreiber, Maria L Moretti, José L Proença-Modena, Mônica Alves

**Affiliations:** 1 Department of Ophthalmology and Otorhinolaryngology, School of Medical Sciences, University of Campinas, Campinas 13083-887, Brazil; 2 Center for Molecular Biology and Genetic Engineering, University of Campinas, Campinas 13083-887, Brazil; 3 Laboratory of Emerging Viruses, Department of Genetics, Microbiology, and Immunology, Institute of Biology, University of Campinas (UNICAMP), Campinas 13083-887, Brazil; 4 Autoimmune Research Laboratory, Department of Genetics, Microbiology, and Immunology, Institute of Biology, University of Campinas (UNICAMP), Campinas 13083-887, Brazil; 5 Laboratory of Aging Biology, Department of Biochemistry and Tissue Biology, Institute of Biology, University of Campinas (UNICAMP), Campinas 13083-887, Brazil; 6 Department of Clinical Pathology, School of Medical Sciences, FCM, University of Campinas (UNICAMP), Campinas 13083-887, Brazil; 7 Department of Internal Medicine, School of Medical Sciences, FCM, University of Campinas (UNICAMP), Campinas 13083-887, Brazil; 8 Laboratory of Electrophysiology, Neurobiology and Behavior, University of Campinas (UNICAMP), Campinas 13083-887, Brazil; 9 Biodiversity Research Center, Federal University of Roraima, Roraima 69310-000, Brazil; 10 Experimental Medicine Research Cluster (EMRC), University of Campinas (UNICAMP), Campinas 13083-887, Brazil

**Keywords:** Coronavirus, COVID-19, eye infections, viral

## Abstract

In this cross-sectional study, we investigate the presence of Severe Acute Respiratory Syndrome Coronavirus 2 Ribonucleic Acid (SARS-CoV-2 RNA) in the tears of hospitalized COVID-19 patients. After laboratory confirmation of SARS-CoV-2 infection by reverse transcription polymerase chain reaction (RT-PCR) analysis, tear samples from both eyes of each patient were collected using conjunctival swab for RT-PCR. Detailed demographic profile, systemic and ocular symptoms, comorbidities, clinical, ancillary, and ocular manifestations were evaluated. Of the 83 patients enrolled in the study, 7 (8.43%) had SARS-CoV-2 RNA detected in the tear samples. Neutrophils’ count, C-reactive protein, and D-dimer were higher in patients with SARS-CoV-2 detected in tears than in patients without virus in ocular surface samples. One patient with SARS-CoV-2 in tears showed mild ocular eyelid edema, hyperemia, and chemosis. No relevant ocular manifestations were detected in the other patients. Although the levels of viral RNA on ocular surface samples were low for most patients (5/7), with positivity only for gene *N* and CT higher than 30, two patients were positive for all viral targets tested (*N*, *E*, and *RpRd*), with viral load near 1 × 10^5^ ePFU/mL, indicating that the ocular transmission of SARS-CoV-2 is a possibility that needs to be considered, especially in the hospital environment. Further studies need to be conducted to demonstrate whether infective viral particles could be isolated from tears.

## Impact statement

In this manuscript, we aimed to investigate the presence of SARS-CoV-2 RNA in tears and ocular surface of COVID-19 hospitalized patients in our tertiary hospital during the disease outbreak. Besides efforts and many advances in the COVID-19 research around the world, the role of ocular surface, as a source of contamination, and the potential risk for heath and eyecare professionals, who would be infected during close contact with patients, remain not entirely understood. Thus, in this study, we evaluated and compared systemic and ocular data in a sample of hospitalized COVID-19 patients, correlating the positivity for RT-PCR for SARS-CoV-2 in ocular surface with all clinical and ocular parameters. Ocular and nasopharynx samples along with data were collected in a considerable number of hospitalized patients in different stages of infection.

## Introduction

COVID-19 is an emerging disease characterized by the presence of fever, fatigue, and myalgia that can progress to severe symptoms, such as dyspnea, hypoxia, respiratory failure, shock, and multiple organ dysfunction.^1,2^ Although ocular manifestations are not frequently observed, some studies have reported patients with conjunctival hyperemia, chemosis, and epiphora among COVID-19 patients.^3^

Around the world, more than 150 million cases have been reported so far.^4^ Brazil is one of the most affected countries, with more than 14 million cases registered and over 400,000 deaths until the end of April 2021.^5^

This disease is caused by a new member of the genus *Betacoronavirus*, named Severe Acute Respiratory Syndrome Coronavirus 2 (SARS-CoV-2).^6^ The transmission of SARS-CoV-2 occurs through respiratory droplets during sneezing, coughing, speaking, or by direct contact with mucous membranes. This virus can also be transmitted by fomites when respiratory secretions or droplets from infected individuals can contaminate objects or surfaces or airborne when aerosols are generated during medical procedures.^1^ Besides, SARS-CoV-2 has been detected in other biological samples, such as feces, urine, tears, and blood. The possibility of transmission during the incubation period or by asymptomatic individuals can contribute to the rapid spread of SARS-CoV-2 worldwide.^7^

COVID-19 diagnosis is based on detecting viral RNA by Reverse Transcription Polymerase Chain Reaction (RT-PCR) in nasopharyngeal swabs, a non-invasive method but uncomfortable approach.^8^ Moreover, false-negative results are not uncommon, as observed in some patients with a computed tomography (CT) scan of the chest suggestive of viral pneumonia.^9^ Thus, an accessible source of clinical samples, such as the conjunctival mucosa, could be useful for SARS-CoV-2 detection, since this virus can be detected in the tears of a significant proportion of COVID-19 patients.^10,11^ Also, ophthalmologists routinely work very close to the patient during eye care exams and treatment, increasing the chance of transmission to these health professionals. Indeed, a large number of ophthalmologists have been infected during the COVID-19 pandemic.^10^ Therefore, it is crucial to understand the fundamental frequency of SARS-CoV-2 detection in the tears of COVID-19 patients and to characterize the risk factors for viral shedding in ocular secretions.

In the present study, we evaluated the presence of SARS-CoV-2 RNA in the tears of patients with COVID-19. We also analyzed clinical, demographic, and laboratory results to search for risk factors for viral detection in tears.

## Materials and methods

From 7 July to 11 August 2020, 83 patients hospitalized in the hospital ward or intensive care at the Hospital de Clínicas of the University of Campinas (Unicamp) were included in this cross-sectional study. The inclusion criteria consisted of the diagnosis of COVID-19 confirmed by identifying the SARS-CoV-2 virus using the RT-PCR method of samples collected through nasopharyngeal and oropharyngeal swabs. Another confirmation of positive PCR result was performed during hospitalization for the patient’s inclusion in the study, regardless of the presence of symptoms and/or severity of the systemic picture. Patients without laboratory confirmation of the disease, despite clinical suspicion, were excluded from the study. On the other hand, asymptomatic patients hospitalized for other diseases had the nasopharyngeal RT-PCR collected, as a hospital protocol. If the result was positive, they were also included in the study, since they had the potential to transmit the disease.

The study has approval by the Ethics Committee of the Unicamp (IRB number 32179720.3.0000.5404) and was conducted according to the Declaration of Helsinki. All patients invited to participate in the study provided written consent for data and ocular swab collection.

### Study design

General information, comorbidities, and systemic and ophthalmological clinical manifestations were obtained from medical records. A specific questionnaire was provided to collect information about the profession, city of residence, education, recent travel history, onset, and types of symptoms, including ophthalmic ones.

Objective data consisted of clinical parameters at sample collection, such as respiratory rate and need for mechanical ventilation. The most recent laboratory tests were also recorded as serum hemoglobin, platelets, total leukocytes, neutrophils, C-reactive protein, lactate dehydrogenase, creatine, and D-dimer. Patients submitted to chest CT had their reports analyzed to classify the involvement of the lung parenchyma as absent, mild, moderate, or severe. All patients underwent an eye evaluation before sample collection. It consisted of directed anamnesis and ectoscopy of the eyes and orbits before and after topical instillation of 1% sodium fluorescein eye drops. Conjunctival hyperemia, chemosis, eyelid edema, eye discharge, and keratitis were investigated and graded.

This study’s design is in line with the SARS-CoV-2 research project proposed by the World Health Organization.^12^ It follows the precepts of its protocol, with the aim that its results and data can be used in future studies with a greater scope and thus contribute to building a more comprehensive and meaningful understanding of SARS-CoV-2 infection.

### Tear collection

Conjunctival sample collection consisted of smearing the lower fornix of the conjunctiva in both eyes with a swab, without topical anesthesia. Each patient had only one sample collected, corresponding to the conjunctival smear performed in both eyes. The material was then placed in a flask with PrimeStore® Molecular Transport Medium and stored at room temperature for a maximum period of seven days. It was sent to the laboratory to detect the SARS-CoV-2 virus by the RT-PCR method.

### SARS-CoV-2 detection in tears

The genomic RNA of each sample was extracted by the Extracta kit – RNA and viral DNA – Locus using an automated extraction instrument Thermo Scientific™, KingFisher™, Flex Purification according to the manufacturer’s instructions. The detection of SARS-CoV-2 by qPCR was amplified using the GeneFinder™ COVID-19 PLUS RealAmp Kit according to the manufacturer’s instructions. Thermal cycle parameters used were 50°C for 20 min, 95°C for 5 min, followed by 45 cycles of 95°C for 15 s, and 58°C for 60 s using the Applied Biosystems® 7500 Fast Real-Time PCR System equipment.

### SARS-CoV-2 quantification in tears

The viral load of SARS-CoV-2 was determined by TaqMan one-step quantitative reverse transcriptase PCR (qRT-PCR) following the Charité protocol.^13^ We used this protocol to amplify and quantify the gene *E* of SARS-CoV-2 after comparison with a standard curve produced using serial 10-fold dilutions of SARS-CoV-2 RNA. All results were expressed on a log10 scale as viral RNA equivalents per milliliter.

### Statistics

Exploratory data analysis was performed through summary measures (mean, standard deviation, minimum, median, maximum, frequency, and percentage). Comparison of continuous variables was performed using the Student’s t-test or Mann–Whitney U test, and categorical variables were compared using the x^2^ test or Fisher’s exact test when appropriate. The level of significance was 5%. The analyses were performed using the computer program SAS System for Windows (Statistical Analysis System), version 9.4. (SAS Institute, Inc, Cary, NC, USA).

## Results

Eighty-three COVID-19 hospitalized patients were included in this cross-sectional study. Besides having positive nasopharyngeal RT-PCR results, only 8.43% (7/83) were found positive in tear samples. Most tears (5/7) had a low viral load, with Cts values higher than 30 (average of 33.85), and positivity only for the gene *N* (nucleoprotein) using the Gene Finder protocol (Table 1). However, two tear samples were positive for all SARS-CoV-2 targets, with low Cts values (average of 22.89 for *E*; 25.97 for *N*; and 23.23 for *RpRd*). Thus, to have a more accurate measure of viral load, the RNA viral levels were determined by comparison with a standard curve using the Charité protocol for gene *E* amplification. As expected, we were able to determine the viral load only in the two tear samples with low Cts values. The mean of viral load in tears was 1.2 × 10^5^ PFU Eq/mL. We believe that the high viral load of SARS-CoV-2 in the tears suggest that SARS-CoV-2 can be able to replicate in ocular surface.

**Table 1.** Cycle threshold values (Cts) averages from positive qRT-PCR ocular swab samples.

Gene finder protocol				Charité protocol	
Patient	Gene *E*	Gene *N*	Gene *RpRd*	Gene *E*	Quantity (PFU Eq/mL)
1	>44	33.02	>44	>44	N/D
2	>44	37.29	>44	>44	N/D
3	>44	36.11	>44	>44	N/D
4	>44	31.96	>44	>44	N/D
5	21.12	27.04	21.72	36.10	1.34 × 10^5^
6	>44	30.9	>44	>44	N/D
7	24.67	24.9	24.75	35.76	1.06 × 10^5^

Gene *E*: envelope; Gene *N*: nucleoprotein; Gene *RdRp*: RNA-dependent RNA polymerase; N/D: non detected; PFU Eq/mL: plate forming units from viral RNA equivalents per milliliter.

Table 2 demonstrates the demographic and clinical data of all patients included in this study. Although ocular manifestations were not more frequently detected in those patients, the proportion of men and the levels of neutrophils, C-reactive protein, and D-dimer were higher in patients with SARS-CoV-2 detected in tears than in the patients without virus in ocular surface samples. Differences in the other parameters were not statistically significant. Asymptomatic patients represented 42.86% of the positive cases and 17.11% of the negative cases.

**Table 2.** Demographic and clinical data of COVID-19 patients.

Variables	*Negative ocular RT-PCR to SARS-CoV-2 (n = 76)*	*Positive ocular RT-PCR to SARS-CoV-2 (n = 7)*	*p-Values*
Gender (male/female)	41 (53.95%) / 35 (46.05%)	5 (71.43%) / 2 (28.57%)	0.01^†^
Age (years)	56.45 ± 13.97 (53.25–59.64)	57.71 ± 16.25 (42.69–72.74)	0.84^‡^
Days since COVID-19 symptoms	15.12 ± 12.65 (11.8–18.45)	21.33 ± 7.77 (2.04–40.63)	0.10^‡^
Days since nasopharyngeal RT-PCR	5.18 ± 4.65 (4.16–6.2)	3.57 ± 2.82 (0.9–6.17)	0.39^‡^
Respiratory rate (breaths per minute)	21.4 ± 4.317 (20.2–22.61)	25 ± 5.66 (17.98–32.02)	0.16^‡^
Need for mechanical ventilation	3 (3.95%)	1 (14.29%)	0.008^†^
Patients at intensive care unit	9 (11.84%)	1 (14.29%)	0.83^‡^
Hemoglobin (g/dL)	12.12 ± 2.262 (11.6–12.63)	13.4 ± 2.51 (11.08–15.72)	0.09^‡^
Platelets (units × 10^3^/µL)	251 ± 113 (225–277)	276 ± 176 (113–440)	0.85^‡^
Neutrophils (units/µL)	5575 ± 3738 (4677–6473)	9107 ± 3829 (5089–13,125)	0.03^†^
C-reactive protein (mg/L)	54.14 ± 54.17 (40.71–67.56)	149.5 ± 142.8 (0–299.3)	0.01^‡^
D-dimer (ng/mL)	1918 ± 2752 (1231–2606)	7287 ± 9056 (0–18,532)	0.04^‡^
LDH (units/L)	266.5 ± 102 (237.9–295.2)	359.8 ± 109.6 (185.3–534.2)	0.09^‡^
Creatinine (mg/dL)	1.34 ± 1.62 (0.96–1.71)	1.12 ± 0.83 (0.29–1.95)	0.72^‡^
CT moderate to severe	23 (30.26%)	2 (28.57%)	0.87^†^

Note: Data are expressed in mean ± standard deviation (95%CI) or frequency (%).

^†^Chi-square test.

^††^Student’s t-test.

RT-PCR: reverse transcription polymerase chain reaction; SARS-CoV-2: Severe Acute Respiratory Syndrome Coronavirus 2; COVID-19: Coronavirus disease 2019; LDH: lactate dehydrogenase; CT: computed tomography.

Detailed analysis of ocular manifestations is displayed in Table 3. Interestingly, 23.64% of negative ocular RT-PCR patients referred to at least one ocular symptom. On the other hand, none of the seven positive ocular RT-PCR patients had any ocular complaints. One of these seven patients denied ocular symptoms when asked, but an ophthalmologic examination showed mild ocular eyelid edema, hyperemia, and chemosis in both eyes.

**Table 3.** Ocular manifestations of COVID-19 patients.

Variable	*Negative ocular RT-PCR to SARS-CoV-2 (n = 76)*	*Positive ocular RT-PCR to SARS-CoV-2 (n = 7)*
Ocular symptoms^†^		
No symptom	58 (76.32%)	7 (100%)
Irritation	0	0
Itching	5 (6.58%)	0
Pink eye	2 (2.63%)	0
Tearing	3 (3.95%)	0
Ocular signs†		
Hyperemia (grade 0–4)	Grade 0: 72 (4.73%)/ Grade1: 3 (3.95%)/Grade 2: 0/ Grade 3:0/ Grade 4: 1(1.32%)	Grade 0: 6 (85.72%)/ Grade1: 0 / Grade 2: 1 (14.28%)/ Grade 3: 0/ Grade 4: 0
Chemosis	1.32%	1 (14.29%)
Eyelid edema	0%	1 (14.29%)
Discharge	1.32%	0
Keratitis	0%	0

Note: Data expressed in mean ± standard deviation (95% CI) or frequency (%)

^†^Chi-square test.

^††^Student’s t-test.

RT-PCR: reverse transcription polymerase chain reaction; SARS-CoV-2: Severe Acute Respiratory Syndrome Coronavirus 2; COVID-19: Coronavirus disease 2019.

Tables 4 and 5 demonstrate the primary demographic, clinical features, and laboratory data of the seven patients tested positive for SARS-CoV-2 on RT-PCR ocular samples.

**Table 4.** Demographic and clinical data of patients tested positive for SARS-Cov-2 on RT-PCR ocular samples.

Patient	1	2	3	4	5	6	7
Gender	Female	Female	Male	Male	Male	Male	Male
Age (years)	47	70	79	58	31	68	51
Days since COVID-19 symptoms onset	Unknown (asymptomatic)	15	30	19	Unknown (asymptomatic)	Unknown (asymptomatic)	21
Days since positive nasopharyngeal RT-PCR	9	2	5	1	4	1	3
Respiratory rate (bpm)	20	32	25	25	20	23	30
Comorbidities	Malignant tumor	Hypertension, Diabetes	Hypertension, Arrhythmia	No	No	Immunosuppression	No
First symptoms	Asymptomatic	Cough, fever, tiredness	Tiredness, dyspnea	Fever	Asymptomatic	Asymptomatic	Headache

RT-PCR: reverse transcription polymerase chain reaction; COVID-19: Coronavirus disease 2019; bpm: breaths per minute.

**Table 5.** Laboratory data of patients tested positive for SARS-CoV-2 on RT-PCR ocular samples.

Patient	1	2	3	4	5	6	7
Hemoglobin (g/dL)	8.8	15.1	14.9	15.1	15	13.9	11
Platelets (units/µL)	66,000	117,000	332,000	293,000	230,000	611,000	288,000
Leukocytes (units/µL)	4820	15,450	11,940	9590	15,010	9100	15,330
Neutrophils (units/µL)	3370	11,430	10,040	6090	11,460	4910	13,030
LDH (units/L)	299	509	260	–	–	371	–
Urea (mg/dL)	23	80	81	51	32	20	37
Creatinine (mg/dL)	0.37	3.08	0.98	1.13	0.89	0.6	0.8
d-Dimer (ng/mL)	880	22,771	6279	–	–	635	5870

LDH: lactate dehydrogenase.

## Discussion

The role of the ocular surface as a possible portal of entry, a reservoir for replication, and transmission of SARS-CoV-2 has been poorly explored. Recently, it has been suggested that both direct infection of the ocular surface or transmission of the virus through tears down the nasolacrimal duct to infect the nasal epithelium are plausible. Recent data demonstrate that conjunctival explants can be infected. Thus, ocular transmission is likely.^14^

There has been a great deal of interest in the medical society and in the lay press about the association of SARS-CoV-2 with the ocular surface. Some studies have been conducted mainly focusing on coronaviruses’ ability to adhere to ocular surface cells, the presence of coronavirus in tears, and its association of conjunctivitis with COVID-19.^15^

One study collected 64 tear samples using a Schirmer strip from 17 nasopharyngeal PCR confirmed COVID-19 patients and analyzed the tears using PCR to detect viral RNA. Ocular samples were collected during three weeks of the infection in the patients. No virus was grown from the tear samples. No viral RNA could be detected, even from those patients with symptoms of upper respiratory tract infection.^16^ In our study, instead of Schirmer strips, conjunctival swab technique was used, similarly to other published articles.

A study conducted in China analyzed 58 conjunctival swab samples from 21 patients with “common-type” and nine patients with “severe-type” COVID-19. Only two samples of one common-type patient yielded viral RNA upon PCR (3%).^10^

A few studies evaluated the possible relationship between the clinical severity of COVID-19 and the positivity of ocular samples as well as ocular manifestation. Generally, ocular samples show low positivity ranging from 2.7 to 15.6%, and ocular manifestations are rare and mostly related to conjunctival alterations (1.5 to 5.26%).^3^ A cross-sectional Indian study evaluated the presence of SARS-CoV-2 in tears of only moderate to severe COVID-19 patients. Thirty-six patients (48%) had moderate disease, whereas 39 patients (52%) had severe disease, with no ocular involvement in any patient. The results showed a considerably higher positivity than the other studies: in the 75 patients, RT-PCR analysis of tears showed positive results in 18 patients (24%), and 29 of 225 samples (12.9%) showed positive results.^17^

A cross-sectional Italian study also showed a high rate of identification of SARS-CoV-2 on the ocular surface of patients with severe disease. SARS-CoV-2 was found on the ocular surface in 52 of 91 patients with COVID-19 (57.1%; 95%CI: 46.3–67.5%) hospitalized in ICU.^18^

In the present study, the higher serum levels of neutrophils, C-reactive protein, and D-dimer, laboratorial markers of systemic inflammation, in patients with SARS-CoV-2 detected in tears might suggest higher severity to be associated with a higher percentage of conjunctival positivity. In addition, there was a greater need for mechanical ventilation among these patients. Nevertheless, there was no statistically significant difference between the groups in relation to the ICU admission rate and the findings on chest tomography, which are two of the main clinical parameters of COVID-19 severity.

An interesting finding form the study is that, among the patients who were positive, the period of days since first symptoms varied from 15 to 30 days, a period when patients are considered not to be transmitting the virus anymore. This raises the possibility that the more severe patients maintain the virus in the ocular surface for more time. Nevertheless, it must be taken into account that the days that elapsed since the onset of symptoms were based only on information provided by the patients themselves, which may be subject to memory bias and, therefore, overestimated. A Chinese study has shown results contrary to ours in this regard. In their study, the mean time from symptom onset to conjunctival swab sampling of positive cases was significantly less than that of negative cases.^19^

Some limitations might be pointed out. Since tears were collected using PrimeStore® Molecular Transport Medium, which promotes viral inactivation, we were not able to isolate SARS-CoV-2 in Vero cell culture. However, it is important to say that, although we have not analyzed any unequivocal markers of viral replication, the high viral load detectable in two patients suggests that SARS-CoV-2 can replicate on the ocular surface. We were able to collect only one sample from each patient; thus, a natural history of positivity during the disease could not be established. In addition, the time elapsed from the positivity of nasopharyngeal PCR to the collection of ocular material was variable among patients. No nasopharyngeal PCR was collected on the same day of collection of ocular material, which limits the correlation between the two methods of collection. Moreover, this cohort included only hospitalized patients, not representing the broad spectrum of SARS-CoV-2-infected ones. Lastly, we cannot prevent respiratory aerosol contaminating the face both before and during the conjunctival swab. Therefore, no one can be sure that there is no aerosol from the mouth/nose reaching to the ocular surface and contaminating it. It is worth mentioning that, due to the inherent practical difficulties, this type of prevention was not found in any other article that addressed the topic.

The positivity of ocular smears to SARS-CoV-2 in RT-PCR tests found in this study was low. Only two patients had CTs values and a viral load compatible with the viral replication in the ocular surface, suggesting that the presence of viable viral particles in the tears is possible for a very small proportion of the SARS-CoV-2-infected patients. However, most of SARS-CoV-2 tear-positive patients had results compatible with a low viral load, since they were positive only to *N* gene, with CTs higher than 30. Thus, the results indicate that the presence of viable viral particles in the tears may not be true for all positive patients. These results together and inability to isolate SARS-CoV-2, since the sampling and transport were done using an inactivating medium, prevented us from concluding that viable viral particles of SARS-CoV-2 are found in the tears of positive patients, constituting a limitation of the study.

Regarding ocular manifestations, only one of the seven positive ocular RT-PCR patients had ocular signs – eyelid edema, hyperemia, and chemosis in both eyes. However, it should be emphasized that this patient was hospitalized in an Intensive Care Unit, and the ophthalmologic examination was performed 15 days after the beginning of COVID-19 symptoms. Thus, the ocular signs may have no direct correlation with SARS-CoV-2 infection. Hence, it cannot be established whether SARS-CoV-2 colonizes the ocular surface and tear fluid or invades ocular surface cells. As the viral load levels were consistently low, it is plausible to assume a weak potential for contamination from the ocular surface or tear film contact.

## Conclusions

Around 8% of the laboratory-proven hospitalized COVID-19 patients had viral RNA in their conjunctival secretions. Thus, the ocular transmission of SARS-CoV-2 is a possibility that needs to be considered, especially in the hospital environment. According to our statistical analysis, there may be a greater chance of finding the virus in tears of male patients with more severe cases. Further studies need to be conducted to demonstrate whether infective viral particles could be isolated from tears.
